# Prognostic Assessment of HLM Score in Heart Failure Due to Ischemic Heart Disease: A Pilot Study

**DOI:** 10.3390/jcm13113322

**Published:** 2024-06-04

**Authors:** Andrea D’Amato, Paolo Severino, Massimo Mancone, Marco Valerio Mariani, Silvia Prosperi, Lorenzo Colombo, Vincenzo Myftari, Claudia Cestiè, Aurora Labbro Francia, Rosanna Germanò, Nicola Pierucci, Francesca Fanisio, Stefanie Marek-Iannucci, Andrea De Prisco, Gianmarco Scoccia, Lucia Ilaria Birtolo, Giovanna Manzi, Carlo Lavalle, Gennaro Sardella, Roberto Badagliacca, Francesco Fedele, Carmine Dario Vizza

**Affiliations:** 1 Department of Clinical, Internal, Anesthesiology and Cardiovascular Sciences, Sapienza University of Rome, Viale del Policlinico 155, 00161 Rome, Italy; andrea.damato@uniroma1.it (A.D.); paolo.severino@uniroma1.it (P.S.); massimo.mancone@uniroma1.it (M.M.); silvia.prosperi@uniroma1.it (S.P.); colombolorenzo90@yahoo.it (L.C.); vincenzo.myftari@uniroma1.it (V.M.); claudia.cestie@uniroma1.it (C.C.); auroa.labbrofrancia@uniroma1.it (A.L.F.); rosanna.germano@gmail.com (R.G.); nicola.pierucci@uniroma1.it (N.P.); stefanie.marekiannucci@uniroma1.it (S.M.-I.); andrea.deprisco@uniroma1.it (A.D.P.); gianmarco.scoccia@uniroma1.it (G.S.); luciailaria.birtolo@uniroma1.it (L.I.B.); giovanna.manzi@uniroma1.it (G.M.); carlolavalle@yahoo.it (C.L.); gennaro.sardella@uniroma1.it (G.S.); roberto.badagliacca@uniroma1.it (R.B.); dario.vizza@uniroma1.it (C.D.V.); 2 Division of Cardiology, Policlinico Casilino, 00169 Rome, Italy; fanisio.francesca@gmail.com; 3 San Raffaele Cassino, 03043 Cassino, Italy; francesco.fedele@uniroma1.it

**Keywords:** heart failure, ischemic heart disease, acute coronary syndrome, etiology, prognosis, cardiovascular death, heart failure hospitalization

## Abstract

**Background:** Ischemic heart disease (IHD) represents the main cause of heart failure (HF). A prognostic stratification of HF patients with ischemic etiology, particularly those with acute coronary syndrome (ACS), may be challenging due the variability in clinical and hemodynamic status. The aim of this study is to assess the prognostic power of the HLM score in a population of patients with ischemic HF and in a subgroup who developed HF following ACS. **Methods:** This is an observational, prospective, single-center study, enrolling consecutive patients with a diagnosis of ischemic HF. Patients were stratified according to the four different HLM stages of severity, and the occurrence of CV death, HFH, and worsening HF events were evaluated at 6-month follow-up. A sub-analysis was performed on patients who developed HF following ACS at admission. **Results:** The study included 146 patients. HLM stage predicts the occurrence of CV death (*p* = 0.01) and CV death/HFH (*p* = 0.003). Cox regression analysis confirmed HLM stage as an independent predictor of CV death (OR: 3.07; 95% IC: 1.54–6.12; *p* = 0.001) and CV death/HFH (OR: 2.45; 95% IC: 1.43–4.21; *p* = 0.001) in the total population of patients with HF due to IHD. HLM stage potentially predicts the occurrence of CV death (*p* < 0.001) and CV death/HFH (*p* < 0.001) in patients with HF following ACS at admission. **Conclusions:** Pathophysiological-based prognostic assessment through HLM score is a potentially promising tool for the prediction of the occurrence of CV death and CV death/HFH in ischemic HF patients and in subgroups of patients with HF following ACS at admission.

## 1. Introduction

Heart failure (HF) is a puzzling syndrome with a complex pathophysiology. It consists of several signs and symptoms, and originates from an insufficient contractile capacity in the heart, leading to inadequate blood supply for all end organs [1].

HF has a high prevalence worldwide, affecting more than 64 million people. In developed countries, it involves 1–2% of the general population. It has an incidence of 1–20 cases per 1000 person-years, with variations according to geographical areas and populations [2]. The HF age-adjusted incidence is decreasing nowadays in industrialized countries due to the improved management of cardiovascular (CV) disease; nonetheless, the overall incidence continues to increase given the ageing of the population [1,2]. Furthermore, acute HF demonstrates a 1-year mortality of 23.6%, while this figure is 6.4% for chronic HF, regardless of left ventricular ejection fraction (LVEF) [2]. These data result in HF being known as “the non-infectious pandemic of the third millennium”.

HF originates from a combination of several cardiac structural and/or functional alterations. These first result in an inadequate cardiac output and later progress into multisystemic disease, leading to the advanced stages of HF [3,4,5]. Ischemic heart disease (IHD) is the main cause of both acute and chronic HF in industrialized countries [1,2,6]. In the Global Congestive Heart Failure Study [7], 40% of the studied HF population had ischemic HF regardless of LVEF, with the highest estimations in Eastern Europe (57%). Patients presenting with ischemic HF require urgent coronary angiography in case of acute coronary syndrome (ACS). The onset of acute HF during a non-ST-elevation myocardial infarction (NSTEMI) is a very-high-risk factor for an immediate invasive angiography [8]. On the other hand, chronic, progressive, and subclinical multivessel coronary artery disease can lead to HF [9], eventually leading to recurrent hospitalization.

Prognostic models of HF have several limitations [10,11,12,13,14]. A prognostic stratification of patients with HF with ischemic etiology, particularly those with ACS, may be challenging with the most common classification systems [1], such as the New York Heart Association (NYHA) class, and LVEF [12] due to the variability of clinical and hemodynamic status during hospitalization. The prognostic stratification of patients with HF should take into account the multisystemic involvement occurring in this syndrome. We have already proposed a new scoring system named HLM score, based on a deep pathophysiological and multisystemic evaluation, that demonstrated a better prognostic power compared to other classification systems within the HF population [3]. A pathophysiological-based prognostic stratification may also be helpful to guide HF treatment, both in terms of intensity and choice of drugs, in light of the metabolic and multisystemic effects of new HF drugs [15,16]. The correct management of disease-modifying drugs, in addition to coronary artery bypass graft (CABG) surgery or percutaneous coronary intervention (PCI), may play a pivotal role among patients with IHD, since they are at high risk of developing HF.

The aim of this pilot study is to evaluate the prognostic power of the HLM score in a population of patients with ischemic HF, and in a subgroup who developed HF following ACS, in terms of CV death, heart failure hospitalization (HFH) and worsening HF (WHF) events (i.e., urgent ambulatory visit/diuretic dose escalation) at 6-month follow-up.

## 2. Methods

This is an observational, prospective, single-center study, enrolling patients with a diagnosis of ischemic HF who were consecutively admitted, between January and June 2023, to the Department of Clinical, Internal, Anesthesiology and Cardiovascular Sciences at Policlinico Umberto I, Sapienza University of Rome. Inclusion criteria were as follows: (i) written, signed, and dated informed consent; (ii) age above 18 years; (iii) diagnosis of HF according to the guidelines [1]; and (iv) documented acute and/or chronic ischemic heart disease as an etiopathogenetic determinant of HF, according to the guidelines [8,9]. Exclusion criteria were as follows: (i) planned or history of heart transplantation and/or ventricular assist device (VAD); (ii) end-stage kidney failure/dialysis; (iii) any condition limiting life expectancy less than one year; (iv) pregnancy or nursing; or (v) non-compliance with the study protocol.

The following parameters were collected: clinical parameters (past medical history, physical examination, electrocardiogram, arterial blood pressure, NYHA class, HLM score, pharmacological therapy at admission and discharge); echocardiographic parameters (ventricular chambers’ size and function, systolic and diastolic function, valve disease and severity); and laboratory parameters (blood cell count, creatinine, estimated glomerular filtration rate (eGFR), serum electrolytes, aspartate aminotransferase, alanine aminotransferase, alkaline phosphatase, gamma glutamyl transferase, ferritin, transferrin, circulating iron, and total, direct, and indirect bilirubin).

The total population was stratified according to the HLM score [3], which includes four different stages of severity (HLM 1–4), and the occurrence of CV death and HFH, the composite of these, and the need for diuretic escalation/an urgent ambulatory visit due to worsening HF were evaluated in order to test the prognostic power of the HLM score at 6-month follow-up. A sub-analysis was performed including only patients who developed HF following ACS at admission.

Data were collected in a dedicated Excel database. The study was conducted according to the Helsinki Declaration. The study protocol was approved by the Ethical Committee of Policlinico Umberto I, Sapienza University of Rome (rif.7068, approved on 8 May 2023).

### 2.1. The HLM Score

The HLM score was based on the HLM classification, considering each value of H, L and M as a numerical variable (Table 1) [3].

The coefficient linear combination (multiplied by 10 and then rounded to the nearest integer) obtained from the Cox PH model for the hazard of the composite and each single outcome was used. The resulting score was as follows:

HLM score = 2H + 3L + 1M

The corresponding HLM stages were as follows:

- HLM score 2–6: HLM-1.
- HLM score 7–11: HLM-2.
- HLM score 12–16: HLM-3.
- HLM score 17–20: HLM-4.

The risk stages were designed to have a good performance in stratifying patients according to their risk and to guarantee an adequate number of subjects in each class (at least 10% of the total).

### 2.2. Statistical Analysis

The normal distribution of variables was assessed with the Shapiro–Wilk test. Continuous variables were expressed as the mean and standard deviation, whereas median and interquartile range were used for non-normally distributed data. Categorical data were described as the number and percentage. Student’s *t*-test, Mann–Whitney test, the χ^2^ test, and the Fisher exact test were used for comparisons, as needed. For comparisons among more than 2 groups, the χ^2^ test was used for categorical variables, the Kruskal–Wallis test was used for non-normally distributed data, and the analysis of variance (ANOVA) was used for continuous variables. The Kaplan–Meier method was used to estimate cumulative event rates at different HLM stages in the overall population and in patients with ACS at presentation. Differences in each group were compared using log-rank tests. The Cox regression hazard model was performed to obtain the hazard ratios (HRs) for the associations among HLM classification and clinical endpoints. For all tests, a *p*-value < 0.05 was considered statistically significant.

The statistical analysis was performed using SPSS version 27.0 for Mac (IBM Software, Inc., Armonk, NY, USA).

## 3. Results

A total of 146 consecutive patients were enrolled in the study. The mean age of the study population at baseline was 73 years (±13). Of the patients, 110 (75.3%) were male. The mean LVEF was 32% (±15). The mean eGFR was equal to 61.8 mL/min/1.73 m^2^ (±38). The baseline features of the total population according to each severity stage of the HLM score are listed in Table 2. Three patients died during the hospitalization: one due to CV causes (cardiogenic shock) and two due to non-CV causes (intracranial bleeding and pneumonia, respectively). The discharge therapy regarding the disease-modifying drugs for HF is presented in Table 3. Patients were followed up for 6 months.

The occurrence of each outcome and the composite of CV death and HFH at 6 months in the total population, according to each HLM stage, are presented in Table 4. Among the different HLM stages, statistically significant differences were reported regarding CV death (*p* = 0.013) and the composite of CV death and HFH (*p* = 0.006). No statistically significant differences were reported in terms of HFH (*p* = 0.29), urgent ambulatory visits (*p* = 0.47), or the need for loop diuretic escalation (*p* = 0.47).

**Figure 1:**
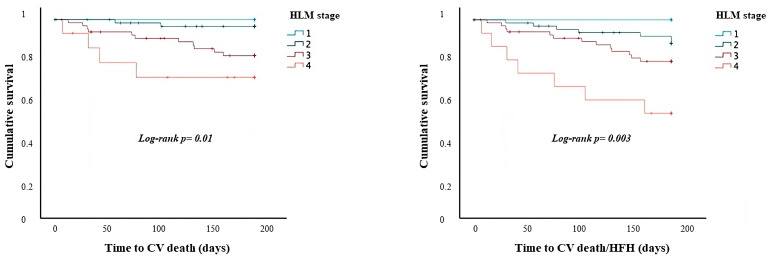
Survival analysis regarding the occurrence of CV death and the composite of CV death and HFH according to each HLM stage in the total population. *CV: cardiovascular; HFH: heart failure hospitalization*.

The Kaplan–Meier survival analysis demonstrated that the HLM stages predict the occurrence of CV death (log-rank *p* = 0.01) and CV death/HFH (log-rank *p* = 0.003) (Figure 1) at 6-month follow-up. The Cox regression analysis confirmed HLM stage as an independent predictor of CV death (OR: 3.07; 95% IC: 1.54–6.12; *p* = 0.001), and the same was found for the composite of CV death/HFH (OR: 2.45; 95% IC: 1.43–4.21; *p* = 0.001) at 6-month follow-up.

Regarding the subgroup of patients who were admitted due to HF following ACS, the occurrence of each outcome and the composite of CV death/HFH at 6 months according to HLM stage is presented in Table 5. Among the HLM stages, statistically significant differences were reported in terms of CV death and CV death/HFH (*p* = 0.013 and *p* = 0.05, respectively). No statistically significant differences were reported in terms of the other outcomes (Table 5).

**Figure 2:**
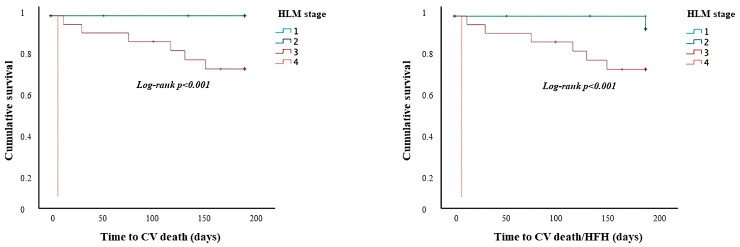
Survival analysis regarding the occurrence of CV death and the composite of CV death/HFH according to HLM stage in the subgroup of patients with HF following ACS at admission. *CV: cardiovascular; HFH: heart failure hospitalization; ACS: acute coronary syndrome*.

The Kaplan–Meier survival analysis demonstrated that HLM stage predicts the occurrence of CV death (log-rank *p* < 0.001) and the composite of CV death/HFH (log-rank *p* < 0.001) at 6-month follow-up in patients with HF following ACS at admission (Figure 2).

## 4. Discussion

HF is known to be a complex clinical syndrome that requires a holistic approach regarding patient care. The current management strategy of HF is “cardiocentric” and usually based on rough parameters, such as LVEF and symptoms [12,14,17,18]. Recently, the definition of HF has been evolving with the introduction of new concepts. The introduction of clinical phenotypes overcomes the definition based only on LVEF, offering practical guidance for cardiologists concerning the management of HF [19,20]. Despite the usefulness of clinical phenotypes, they sometimes remain superficial, as they neglect the concrete pathophysiological rationale behind clinical aspects while focusing on signs and symptoms [14]. A more comprehensive approach is needed regarding the assessment of the syndrome in order to overcome the initial narrow vision surrounding this disease and achieve a more global approach [3].

Regarding the pathophysiology of HF, it is a progressive syndrome starting from the heart that then involves progressively other organs potentially leading to multisystemic organ dysfunction and eventually death [3]. The multisystemic effects of HF resemble the cancerous spread observed in malignancy [3]. Therefore, HLM score has close similarities with the TNM classification of cancer used in oncology [3]. The HLM scoring system is thought to overcome the limitations of classic prognostic models. The scale is a holistic approach, created with the aim evaluating patients and targeted therapies globally beyond the assessment of symptoms and/or LVEF, combining clinical, laboratory, and instrumental parameters, and focusing on the pathophysiological background of HF. We have already demonstrated the prognostic power of the HLM score compared to other classification systems [3].

In this study, the prognostic discrimination offered by the HLM score has been demonstrated in a population of ischemic HF patients and in a subgroup of patients presenting with HF following ACS at admission, highlighting the importance of a pathophysiologically based prognostic stratification according to specific ischemic etiology and acute ischemic presentation. Given its evolving behavior, according to clinical evolution and therapeutic/interventional approach, the latter may make the prognostic stratification of patients particularly challenging. The severity stages of the HLM scoring system define the progressive involvement of the heart, lungs, and peripheral organs in terms of severity, as well as the progressive spread of the syndrome within the body, with the highest severity stage being associated with the worst prognosis. Our results demonstrated that the HLM score represents a valid prognostic tool for the prediction of the composite of CV death/HFH (*p* = 0.003) and CV death alone (*p* = 0.01) in patients with ischemic HF. Cox regression analysis confirmed HLM stage as an independent predictor of CV death (OR: 3.07; 95% IC: 1.54–6.12; *p* = 0.001) and the composite of CV death/HFH (OR: 2.45; 95% IC: 1.43–4.21; *p* = 0.001) in the total population with HF due to IHD. Moreover, in the subgroup of patients admitted due to HF following ACS at admission, the HLM score potentially preserves its prognostic power in terms of the composite of CV death/HFH (*p* < 0.001) and CV death (*p* < 0.001) prediction.

This aspect further underlines the importance of redefining HF as a multisystemic syndrome rather than a simple heart disease, with pulmonary involvement and multisystemic end-organ damage severely impacting patient prognosis at each stage of disease [21,22,23]. The progressive risk of adverse outcomes according to increasing HLM severity stage sheds light on the importance of pathophysiology over clinical presentation in HF. HLM’s prognostic stratification overcomes the issues of clinical and simplistic models based on the modality of symptom onset (i.e., acute vs. chronic), symptom entities (i.e., NYHA class), and clinical phenotypes, and it contextualizes the role of echocardiographic (i.e., LVEF, TAPSE), pulmonary, and peripheral organ parameters. The pathophysiological background behind HLM’s prognostic stratification retrieves the results of the ESC-EORP-HFA Heart Failure Long-Term Registry [24], highlighting the utility of congestion and perfusion assessment at admission and discharge to provide relevant information to manage HF patients. The presence of hypoperfusion at admission is associated with increased in-hospital adverse outcomes, as well as the presence of in-hospital and residual congestion at discharge with increased mortality at 1-year follow-up [24]. In this regard, Espinosa et al. demonstrated that the presence of congestion and hypoperfusion at emergency department admission is an important marker of short-term adverse outcomes [25].

HF often arises as a consequence of IHD, as atherosclerotic coronary artery disease results in insufficient blood flow to the myocardium. Chronic ischemia, in fact, contributes to myocardial injury, initiating a cascade of events that may culminate in HF. HF following a myocardial infarction manifests in approximately one out of every three patients within a year, and the presence of HF is indicative of markedly elevated long-term mortality [26]. HF with reduced ejection fraction (HFrEF) and HF with mildly reduced ejection fraction (HFmrEF) have been found to be the prevailing subtypes of HF after myocardial infarction [27], although the literature data are ambiguous regarding the predominance of the different HF subcategories. Regardless of LVEF, HF related to IHD is marked by a high risk of decompensation in the first year after the acute myocardial infarction [28].

From a pathophysiological point of view, the onset of HF following a myocardial infarction stems from the altered ventricular remodeling (AVR) of the left ventricle. AVR subsequent to myocardial infarction is marked by the heart’s adaptive changes, mainly concerning ventricular dimensions and performance. This is due to mechanical and neurohormonal factors, as well as the impact of ischemia/reperfusion injury and energy metabolism changes [29]. The loss of functioning viable cardiomyocytes results in myocardial necrosis, which leads to a series of intracellular signaling processes that set in motion and subsequently regulate reparative alterations. These changes encompass dilation, hypertrophy, and the development of a distinct collagen scar [30]. Metabolic alterations result in the hindrance of substrate switching to fatty acids, combined with compromised mitochondrial function. This impedes the oxidation of the augmented lipid load, contributing to contractile dysfunction and the initiation of apoptosis [31]. Furthermore, the inadequacy of coronary circulation to meet cardiomyocytes’ metabolic demand, attributed to the imbalance of mechanisms involved in coronary blood flow regulation, such as ion channel dysfunction, results in coronary microvascular dysfunction [32,33]. This dysfunction leads to the onset of hypoxia, fibrosis, and tissue necrosis, potentially causing a decline in myocardial function.

Our pilot study has several limitations. While the HLM score was tested on a particular population (i.e., ischemic HF) and on a specific subset of patients (i.e., patients with HF following ACS), confirmation on a larger population and a longer follow-up are required to confirm these preliminary observations. Several parameters that may further improve prognostic power (i.e., biomarkers, invasive hemodynamic parameters) have not yet been included in the HLM score.

## 5. Conclusions

HF is a complex disease with a multifaceted pathophysiology [3,4]. The cardiocentric view of HF has several limitations and it based on a rough and superficial evaluation of patients [14]. A transition to a more comprehensive and global approach to HF patients is required, since HF is a progressive disease that predisposes patients to adverse events [3,4,12,13]. The pathophysiologically based prognostic HLM score may potentially address this complexity. IHD is the main etiology of HF, accounting for most HF cases, particularly in industrialized countries [1,2,8,9]. We demonstrated that HLM stages may represent independent predictors of the composite of CV death/HFH and CV death in ischemic etiology HF. Moreover, its prognostic power is potentially maintained in the subgroup of patients with HF following ACS at admission, whose prognostic assessment may be more challenging due to the quick clinical and hemodynamic evolution of the condition, as well as the effects of medical therapy and revascularization. These preliminary results on ischemic HF patients strengthen our previous observation on the general HF population [3], suggesting HLM score as a potentially interesting prognostic tool for HF.

In future, the HLM score, focusing on the pathophysiological background of HF, may be useful to help clinicians to define a personalized, patient-tailored therapeutic regimen.

## Figures and Tables

**Table 1:** **HLM classification**. Progressive stages of severity according to each parameter included in the HLM classification [3].

Heart (H)	Lungs (L)	Malfunction of Other Organs (M)
**H1:** Diastolic dysfunction and/or presence of structural cardiac damage * in absence of LV systolic dysfunction (LVEF ≥ 50%)	**L0:** Absence of any lung involvement	**M0:** Absence of malfunction of other organs **
**H2:** LV systolic (LVEF < 50%) or diastolic dysfunction with structural damage without LV dilation	**L1:** Hemodynamic lung involvement, assessed by CXR, and/or sPAP ≥ 35 mmHg at rest, assessed by TTE, with absence of clinical signs of lung congestion	**M1:** Presence of single end-organ damage (except heart and lungs)
**H3:** LV dilation, structural cardiac damage with systolic (LVEF < 50%) or diastolic dysfunction, or right ventricular systolic dysfunction (TAPSE < 17 mm)	**L2:** Clinical signs and symptoms of lung congestion assessed by physical examination (crepitation, raised jugular venous pressure, orthopnea, dyspnea, necessity of supplemental oxygen due to cardiac causes) and increase in left ventricular filling pressure, assessed by echocardiographic evaluation and, if feasible, by right heart catheterization	**M2:** Presence of two instances of distinct end-organ damage (except heart and lungs)
**H4:** Biventricular systolic dysfunction (LVEF < 50% and TAPSE < 17 mm)	**L3:** “Cardiac lung”, defined by arterialization of pulmonary vasculature, with post-capillary pulmonary hypertension (type II) and necessity of supplemental oxygen at discharge due to cardiac causes, despite use of congestion relief therapy and absence of congestion	**M3:** Presence of ≥3 instances of end-organ damage (except heart and lungs)

LV: left ventricle; LVEF: left ventricular ejection fraction; TAPSE: tricuspid annular plane systolic excursion;; CXR: chest X-rays; sPAP: systolic pulmonary arterial pressure; TTE: transthoracic echocardiography; GFR; glomerular filtration rate; AST: aspartate aminotransferase; ALT: alanine aminotransferase;; CT: computed tomography; MRI: magnetic resonance imaging. * Structural damage is defined by at least one of the following: abnormal wall motion, left ventricular hypertrophy, moderate-to-severe left-sided valvular disease. ** Malfunction of other organs considered as a consequence of HF, defined as follows: GFR < 60 mL/min regarding kidney dysfunction; elevation at least twice as high than normal of at least one parameter among AST/ALT/total bilirubin/gamma-glutamyl transferase/alkaline phosphatase regarding liver dysfunction; hemoglobin < 13 g/dL for men and <12 g/dL for women, regarding anemia; transferrin saturation < 20% with serum ferritin between 100 and 299 ng/mL or serum ferritin < 100 ng/mL alone, regarding iron deficiency; more than 5% edema-free body weight loss during the previous year or less, regarding HF-related cachexia. The Beck Depression Inventory and Cardiac Depression Scale was used to assess HF-related depression and anxiety disorders and CT/MRI to exclude ischemic or hemorrhagic stroke, regarding central nervous system involvement.

**Table 2:** Baseline features of the study population and differences among patients admitted according to the four HLM severity stages at hospital admission.

Variable	Total Population (N = 146)	HLM-1 (N = 4)	HLM-2 (N = 63)	HLM-3 (N = 64)	HLM-4 (N = 15)	*p* Value
**Age, years (SD)**	73 (13)	63 (21)	75 (12)	73 (13)	71 (12)	0.55
**Male gender, *n* (SD)**	110 (75.3)	4 (100)	48 (76.2)	47 (73.4)	11 (73.3)	0.68
**Previous HFH, *n* (%)**	64 (44)	1 (25)	32 (50.8)	25 (39.1)	6 (40)	0.47
**Acute decompensated HF, *n* (%)**	62 (42.5)	2 (50)	21 (33.3)	28 (43.8)	11 (73.3)	0.04
**Acute pulmonary edema, *n* (%)**	9 (6.2)	0 (0)	2 (3.2)	7 (10.9)	0 (0)	0.19
**Cardiogenic shock, *n* (%)**	3 (2.1)	0 (0)	0 (0)	2 (3.1)	1 (6.7)	0.34
**Arterial hypertension, *n* (%)**	116 (79.5)	3 (75)	51 (81)	50 (78.1)	12 (80)	0.98
**Diabetes mellitus, *n* (%)**	53 (36.3)	0 (0)	20 (31.7)	28 (43.8)	5 (33.3)	0.22
**Dyslipidemia, *n* (%)**	96 (65.8)	4 (100)	40 (63.5)	43 (67.2)	9 (60)	0.47
**Family history of CVD, *n* (%)**	38 (26)	3 (75)	12 (19)	17 (26.6)	6 (40)	0.04
**Smoking habit, *n* (%)**	67 (45.9)	2 (50)	31 (49.2)	26 (40.6)	8 (53.3)	0.72
**COPD, *n* (%)**	25 (17.1)	1 (25)	10 (16)	11 (17)	3 (20)	0.953
**Iron deficiency, *n* (%)**	22 (15)	0 (0)	8 (12.7)	8 (12.5)	6 (40)	0.035
**Atrial fibrillation, *n* (%)**	46 (31.5.)	1 (25)	15 (23.8)	23 (36)	7 (46.6)	0.262
**ACS at admission, (%)**	42 (28.8)	1 (25)	18 (28.6)	21 (32.8)	2 (13.3)	0.2
**ICD, *n* (%)**	37 (25.3)	0 (0)	10 (15.9)	18 (28.1)	9 (60)	0.003
**CRT-D, *n* (%)**	11 (7.5)	0 (0)	5 (7.9)	4 (6.3)	2 (13.3)	0.75
**PMK, *n* (%)**	19 (19.2)	0 (0)	6 (9.5)	9 (14.1)	4 (26.7)	0.28
**LVEF, % (SD)**	32 (15)	41.3 (9)	35 (16)	30 (12)	25 (10)	<0.001
**TAPSE, mm (SD)**	18 (5)	21 (11.2)	18 (4)	18 (6.8)	14 (4)	0.09
**LVEDD, mm (SD)**	57.5 (13)	60 (15.2)	55 (13)	59.5 (10)	60 (7)	0.08
**IVS, mm (SD)**	11 (2)	11.5 (1.8)	11 (2)	11 (2)	10 (5)	0.87
**PW, mm (SD)**	10 (1.2)	9 (1)	10 (1)	10 (2)	10 (4)	0.41
**eGFR, mL/min/m^2^ (SD)**	61.8 (38)	85.5 (27)	70 (29.5)	58 (33)	40 (21)	0.003

HFH: heart failure hospitalization; HF: heart failure; CVD: cardiovascular disease; ACS: acute coronary syndrome; COPD: chronic obstructive pulmonary disease; ICD: implantable cardioverter defibrillator; CRT-D: cardiac resynchronization therapy with defibrillator; PMK: pacemaker; LVEF: left ventricular ejection fraction; TAPSE: tricuspid annular plane systolic excursion; LVEDD: left ventricular end-diastolic diameter; IVS: interventricular septum; PW: posterior wall; eGFR: estimated glomerular filtration rate.

**Table 3:** Data regarding discharge therapy for heart failure with disease-modifying drugs in relation to the total population and HLM severity stages.

Variable	Total Population (N = 143)	HLM-1 (N = 4)	HLM-2 (N = 63)	HLM-3 (N = 62)	HLM-4 (N = 14)	*p* Value
**BB, *n* (%)**	139 (97.2)	4 (100)	60 (95.2)	61 (98.4)	14 (100)	0.82
**ACEi/ARBs, *n* (%)**	32 (22.4)	2 (50)	17 (27)	12 (19.3)	1 (7.1)	0.16
**ARNI, *n* (%)**	86 (60.1)	2 (50)	37 (58.7)	34 (54.8)	13 (92.9)	0.12
**SGLT2i, *n* (%)**	72 (50.3)	1 (25)	31 (49.2)	30 (48.4)	10 (71.4)	0.06
**MRAs, *n* (%)**	117 (81.8)	2 (50)	48 (76.2)	55 (88.7)	12 (85.7)	0.24

BB: beta blockers; ACEi: angiotensin-converting enzyme inhibitors; ARBs: angiotensin receptor blockers; ARNI: angiotensin receptor neprilysin inhibitor; SGLT2i: sodium-glucose cotransporter 2 inhibitors; MRAs: mineralocorticoid receptor antagonists.

**Table 4:** Occurrence of each outcome at 6-month follow-up according to HLM stage in the total population.

	Total Population (N = 143)				
Outcome	HLM-1	HLM-2	HLM-3	HLM-4	*p* Value
**HFH, *n* (%)**	0 (0)	5 (7.9)	4 (6.5)	3 (21.4)	0.29
**CV death, *n* (%)**	0 (0)	2 (3.2)	11 (17.7)	4 (28.6)	0.013
**Urgent ambulatory visit, *n* (%)**	0 (0)	12 (19)	9 (14.5)	4 (28.6)	0.47
**Diuretic dose escalation, *n* (%)**	0 (0)	12 (19)	9 (14.5)	4 (28.6)	0.47
**Composite CV death/HFH, *n* (%)**	0 (0)	7 (11.1)	13 (21)	7 (50)	0.006

HFH: heart failure hospitalization; CV: cardiovascular.

**Table 5:** Occurrence of each outcome for patients with heart failure following acute coronary syndrome at admission.

	ACS Population (N = 41)				
Outcome	HLM-1	HLM-2	HLM-3	HLM-4	*p* Value
**HFH, *n* (%)**	0 (0)	1 (5.6)	1 (4.8)	0 (0)	0.9
**CV death, *n* (%)**	0 (0)	0 (0)	6 (28.6)	1 (100)	0.013
**Urgent ambulatory visit, *n* (%)**	0 (0)	3 (16.7)	4 (19)	0 (0)	0.92
**Diuretic dose escalation, *n* (%)**	0 (0)	3 (16.7)	4 (19)	0 (0)	0.92
**Composite CV death/HFH, *n* (%)**	0 (0)	1 (5.6)	6 (28.6)	1 (100)	0.05

ACS: acute coronary syndrome; CV: cardiovascular; HFH: heart failure hospitalization.

## Data Availability

The raw data supporting the conclusions of this article will be made available by the authors on request.
